# New insights into azelaic acid-induced resistance against *Alternaria Solani* in tomato plants

**DOI:** 10.1186/s12870-024-05397-7

**Published:** 2024-07-19

**Authors:** Mostafa Haghpanah, Nadali Babaeian Jelodar, Hamid Najafi Zarrini, Ali Pakdin-Parizi, Ali Dehestani

**Affiliations:** 1 Kohgiluyeh and Boyerahmad Agricultural and Natural Resources Research and Education Center, Dryland Agricultural Research Institute, Agricultural Research, Education and Extension Organization (AREEO), Gachsaran, Iran; 2 Department of Plant Breeding and Biotechnology, Sari Agricultural Sciences and Natural Resources University, Sari, Iran; 3 Genetics and Agricultural Biotechnology Institute of Tabarestan, Sari Agricultural Sciences and Natural Resources University, Sari, Iran

**Keywords:** Defense response, JA/ET pathway, Necrotrophic pathogen, Phytohormone analysis, Systemic acquired resistance

## Abstract

**Background:**

The effect of azelaic acid (Aza) on the response of tomato plants to *Alternaria solani* was investigated in this study. After being treated with Aza, tomato plants were infected with *A. solani*, and their antioxidant, biochemical, and molecular responses were analyzed.

**Results:**

The results demonstrated that H_2_O_2_ and MDA accumulation increased in control plants after pathogen infection. Aza-treated plants exhibited a remarkable rise in peroxidase (POD) and catalase (CAT) activities during the initial stages of *A. solani* infection. Gene expression analysis revealed that both Aza treatment and pathogen infection altered the expression patterns of the *SlNPR1*, *SlERF2*, *SlPR1*, and *SlPDF1*.2 genes. The expression of *SlPDF1*.2, a marker gene for the jasmonic acid/ethylene (JA/ET) signaling pathway, showed a remarkable increase of 4.2-fold upon pathogen infection. In contrast, for the *SlNPR1*, a key gene in salicylic acid (SA) pathway, this increased expression was recorded with a delay at 96 hpi. Also, the phytohormone analysis showed significantly increased SA accumulation in plant tissues with disease development. It was also revealed that tissue accumulation of JA in Aza-treated plants was increased following pathogen infection, while it was not increased in plants without pathogen inoculation.

**Conclusion:**

The results suggest that the resistance induced by Aza is mainly a result of modulations in both SA and JA pathways following complex antioxidant and molecular defense responses in tomato plants during *A. solani* infection. These findings provide novel information regarding inducing mechanisms of azelaic acid which would add to the current body of knowledge of SAR induction in plants as result of Aza application.

## Background

Tomato (*Solanum lycopersicum* L.) is considered the most important horticultural crop in the world due to its high nutritional and economic value [1]. It is cultivated in almost all regions of the world, encountering various biotic and abiotic stresses that can significantly affect growth and productivity [2]. Fungal pathogens are a common cause of several tomato diseases [3]. Early blight, caused by *Alternaria solani*, is a highly destructive disease that affects tomatoes and can reduce total yield by up to 80% [4]. Several control measures, including cultural practices, resistant cultivars, and fungicide applications, have been implemented to inhibit pathogen damage in tomato crops [5]. While certain fungicides can effectively manage this necrotrophic pathogen, their application is limited due to the development of resistance in the fungal population, as well as environmental and health concerns [4, 6]. The exploitation of plants’ natural defense systems has gained more attention recently as a promising approach for controlling and mitigating the adverse effects of diseases by addressing the infection and growth of pathogens [1, 7, 8].

Plants use a variety of defense mechanisms, such as systemic acquired resistance (SAR) and induced systemic resistance (ISR), to inhibit pathogens [9]. It has been revealed that certain natural and synthetic chemicals, commonly referred to as resistance inducers, can activate the plant’s immune system [10–12].

Resistance inducers enhance general resistance of plants to biotic and abiotic stresses by activating various defense mechanisms [6]. Plant-induced resistance is strongly influenced by phytohormones, such as salicylic acid (SA) and jasmonic acid (JA) [13]. In addition, it was suggested that there are some key genes in SA and JA/ET pathways that could be used for plant-pathogen interaction studies. There are several reports of exploiting expression data of *NPR1* and *PR1* genes for the SA pathway and *ERF2* and *PDF1*.2 (*Defensin*) for JA/ET pathway for elucidating plant response to different pathogens. On the other hand, pathogen infiltration activates subsequent immune responses through a sudden increase in reactive oxygen species (ROS) production by the host plant defense machinery [14]. Some biochemical elements, such as microbe-associated molecular patterns (MAMPs) [15], can improve the plant immune system by activating related signaling pathways. Additionally, certain chemicals, such as dicarboxylic acids, can trigger plant defense mechanisms [16].

Azelaic acid (Aza) is a dicarboxylic acid with nine carbon atoms that has recently gained attention as a natural inducer of the plant defense system [17]. Azelaic acid is a derivative of oleic, linoleic, and saturated linolenic acid. However, the major enzymes involved in biosynthetic pathways are not well understood [18]. Jung et al. (2009) demonstrated that Aza induces SAR by increasing the biosynthesis of salicylic acid (SA) in Arabidopsis plant upon bacterial inoculation, resulting in a more robust and faster response to *Pseudomonas syringae* attack, accompanied by higher SA accumulation [19]. Studies conducted by Shah and Zeier (2013) have demonstrated that treatment with Aza leads to an increase in the levels of endogenous SA and enhances the expression of the *PR1* gene in plant tissues upon contact with pathogens [17]. Studies on tobacco plants have indicated that Aza has an inducing effect on the plant immune system by activating genes involved in SAR reactions mainly by increasing the synthesis of hydroxycinnamic acids and related compounds [20].

The use of inducers, such as azelaic acid, can stimulate innate resistance in plants and reduce the damage caused by diseases, without the negative effects of fungicides. However, there has been limited research conducted on the impact of azelaic acid on tomato diseases. Therefore, this study aims to investigate the effect of azelaic acid on the physio-biochemical and molecular responses of a susceptible tomato genotype to *A. solani* infection. The findings of this study would shed light on the current scientific knowledge regarding the role of Aza in SAR induction and plant defense mechanisms. Sophisticated precise application of Aza as a promising resistance inducer would be an environment-friendly alternative to current plant disease controlling strategies.

## Materials and methods

### Plant growth and azelaic acid application

The experiments were conducted as a factorial experiment based on a completely randomized design (CRD) with at least three replicates per treatment (at least six plants for each replication). The experiments were repeated three times. Karoon cultivar (*Solanum lycopersicum* L.) seeds used throughout this study were kindly provided by Falat Iranian Zamin Co, Karaj, Iran. The seeds were sown and grown in 3-liter pots (19 cm) filled with a sterile soil mix consisting of equal volumes of peat, perlite, and coconut peat. The pots were incubated in a growth chamber under controlled conditions with a photoperiod of 12 h of light (560 µmol/m^2^/s), 70% humidity, and a temperature range of 24–27 °C (day/night). They were regularly fertilized with Hoagland’s nutrient solution [21]. Thirty-five-day-old plants (vegetative stage) were sprayed with 1 mM Aza solution (dissolved in 5 mM MES (2-(N-morpholino) ethanesulfonic acid) buffer) [19] until dripping off, while control plants were treated with 5 mM MES (15 ml for each plant). Forty-eight hours after treatment, the plants were inoculated with *Alternaria solani* spores (1.6 × 10^6^) obtained from the microbial culture collection of Tabarestan Agricultural Genetics and Biotechnology Research Institute (Accession No. GABIT-As01).

### Pathogen growth, plant inoculation, and experimental treatments

A spore suspension of *A. solani* at a density of 1.6 × 10^6^ spores per ml was used to infect the plants. Initial pathogenicity tests were performed and infection and disease development were confirmed [22]. The fungal mycelia were gently removed from the surface of five-day-old fungi PDA cultures (pH 6.5). The plates were exposed to 365 nm (black light) for eight hours to induce fungal sporulation. For further analysis, sample collection was conducted at 0, 12-, 24-, 48-, and 96 h post-inoculation (hpi). Four different treatments were included in the study as follows: Control (tomato plants without treatment and inoculation), C+ (tomato plants inoculated with *A. solani*), Aza (tomato plants treated with azelaic acid), and Aza+ (tomato plants pretreated with azelaic acid and inoculated with *A. solani*).

### Disease severity assay

Twenty-one days after inoculation, the percentage of disease symptoms on the randomly selected leaves was estimated for all treatments using ImageJ software [23]. At least 30 plants were investigated for each treatment, and different degrees of disease severity were calculated in the range of zero to five, as outlined in Table 1 [24].

**Table 1:** Symptom ranking scale to assess tomato early blight disease

Rank	Infection percentage and leaf state
0	Healthy green leaves without disease spots
1	Less than 5% of the leaf area had diseased spots
2	Between 6 and 20% of the leaf area had disease spots
3	Between 21 and 40% of the leaf area had disease spots
4	Between 41 and 60% of the leaf area had disease spots
5	More than 60% of the leaf surface was diseased and significantly damaged

### Growth parameters

Shoot fresh weight was measured 21 days after inoculation. The shoots were placed in an oven at 72 °C for 72 h, and the dry weight was measured afterward.

### Biochemical assays

#### Crude enzyme extraction

Crude enzyme extract of tomato leaves was prepared using 1.8 ml of extraction buffer (50 mM Tris-HCl solution with pH 8, Triton X-100 1%, 0.1% mercaptoethanol), which was added to 200 mg of powdered leaf tissue [25]. The solution was centrifuged for 15 min at 15,000 g at 4 °C. The supernatant was used to estimate the total protein content and analyze the activity of different enzymes [26].

#### Total protein content

A 40 µl aliquot of enzyme extract was mixed with 960 µl of Bradford solution. The absorbance of the solution was then read at 595 nm to estimate the total protein content [27]. Bovine serum albumin (BSA) was used as the standard to create the standard curve (R2 = 0.988, Y = 0.0197X + 0.027). The data were then used to determine the activities of and superoxide dismutase (SOD), catalase (CAT), ascorbate peroxidase (APX), and peroxidase (POD) precisely. All enzyme activity and metabolite accumulation assays were conducted using a UV-visible spectrophotometer device (PG Instruments, Model T92 + Double^®^ Beam, England).

#### SOD activity assay

Superoxide dismutase activity was measured according to the method developed by Beauchamp and Fridovich (1971). The reaction mixture (5 ml) containing potassium phosphate buffer (50 mM), EDTA (0.1 µM), methionine (0.013 mM), riboflavin (2 µM), and 50 µl of protein extract was exposed to fairly intense light (300 µmol m^-2^ s^-1^) for 5 min [28]. One enzyme unit in this experiment was estimated to be equivalent to a 50% reduction in NBT optical absorption at 560 nm compared to the control.

#### CAT activity assay

Catalase enzyme activity was measured using the method described by Aebi (1974). The reaction mixture (3 ml) contained sodium phosphate buffer (100 mM, pH 7), H_2_O_2_ (15 mM), and 50 µl of protein extract [29]. Decreasing H_2_O_2_ absorption at 240 nm was considered CAT enzyme activity, and the enzyme unit was estimated using the extinction coefficient (ε = 39.7 M^-1^cm^-1^). CAT activity was defined as unit per minute per milligram of protein.

#### APX activity assay

The reaction mixture consisted of 50 mM phosphate buffer (pH = 7), 0.5 mM ascorbic acid, 0.15 mM hydrogen peroxide, and 100 µl of enzyme extract [30]. Reduced adsorption was measured for 2 min at 290 nm. The enzyme unit was estimated using the extinction coefficient (ε = 2.8 mMol^-1^cm^-1^), APX enzyme activity was defined as unit per minute per milligram of protein.

#### POD activity assay

The guaiacol peroxidase assay was performed using a mixture of 50 mM phosphate buffer (pH 7), 8.26 mM guaiacol, 8.8 mM hydrogen peroxide, and 100 µl of enzyme extract [31]. Absorption changes at 470 nm were observed over two minutes. The enzyme unit was defined based on the extinction coefficient (ε = 25.5 mMol^-1^cm^-1^), and POD enzyme activity was defined as unit per minute per milligram of protein.

#### Lipid peroxidation and H_2_O_2_ assays

About 0.2 gr of powdered tissue was combined with 5 ml of 2% TCA (trichloroacetic acid) and centrifuged at 12,000 g for 15 min. The upper phase was used to calculate the MDA and H_2_O_2_ contents, as described in the following Sects. [14, 32].

MDA was measured using the method described by Ohkawa et al. (1979) with some modifications [33]. One mL of supernatant was blended with four mL of 20% trichloroacetic acid, which contained 0.5% thiobarbituric acid. The mixture was heated for 30 min at 95 °C and then rapidly cooled in an ice bath. After a 10-minute centrifugation at 10,000 g, the concentration of MDA was determined by subtracting the non-specific absorption at 600 nm from the absorption at 532 nm using the extinction coefficient of 156 mM^-1^ cm^-1^.

Hydrogen peroxide was measured using a spectrophotometer after reacting with KI. The reaction mixture consisted of 500 µl of TCA (trichloroacetic acid) supernatant, 500 µl of 0.1 M PBS (potassium phosphate buffer), and 2 ml of potassium iodide (1 M KI). After one hour, the reaction was kept in the dark. The absorbance was measured at 390 nm [34]. The concentration was measured using a standard curve plotted within the range of 100-25000 nmol H_2_O_2_ (Y = 0.4349X + 0.0771, R^2^ = 0.98).

#### Phytohormone analysis

High-performance liquid chromatography (HPLC) technique was used to measure the changes in salicylic acid (SA) and jasmonic acid (JA) levels in tomato leaves. Briefly, one gram of the leaves was powdered using liquid nitrogen and then mixed with 20 ml of 80% methanol containing Diethyldithiocarbamate (3.5 M). The mixes were incubated in the dark for eight hours at 4 °C and then centrifuged at 12,000 g for 20 min at 4 °C. Solvents were evaporated using a speed vacuum system (SPD121P, ThermoSavant, Hastings, United Kingdom) at 25 °C and stored at -20 °C for further analysis. Phytohormone analysis was conducted using an HPLC system equipped with a Surveyor Autosampler, Surveyor LC pump (Thermo Finnigan, Waltham, MA, USA), and a reversed-phase column (ZORBAX 300SB-C18, 2.1 × 150 mm, 3.5 µM; Agilent, Santa Clara, CA, USA). Peaks were identified using analytical grade quality of Salicylic acid and dihydrojasmonic acid standards (Sigma-Aldrich (St. Louis, Missouri, USA)).

### Gene expression analysis

#### RNA isolation and cDNA synthesis

Total RNA was extracted from the plant leaves using Threezol reagent (Riragene, Iran) according to the manufacturer’s instructions. Subsequently, the extracted RNA was treated with DNaseI (Fermentas, Germany) to remove any DNA contaminations. Based on the manufacturer’s protocol, the RevertAid™ Reverse Transcriptase kit (Fermentas, Germany) was used to synthesize cDNA from 1.5 µg of total RNA.

#### qRT-PCR analysis

Specific primers for the *SlNPR1*, *SlERF2*, *SlPR1*, and *SlPDF1.2* genes were used to amplify PCR products (Table 2), and *Actin* gene of *S. lycopersicum* was used as an internal reference. The Maxima SYBR Green/ROX qPCR Master Mix (Thermo Scientific) was used for qRT-PCR reactions. The 15 µl reaction mixture contained 1.0 µl of diluted cDNA sample, 0.3 µM of each forward and reverse primer, and 1× real-time SYBR Green master mix. The cycling temperature conditions included an initial denaturation at 95 °C for 8 min, followed by 40 cycles of 95 °C for 15 s and 60 °C for 30 s. Each sample was quantified in three biological and two technical replicates. Livak and Schmittgen (2001) method (2^−ΔΔCt^) was used to quantify the relative gene expression [35].

**Table 2:** The primer sequences used for qPCR analysis of gene expression in *A. solani*-infected tomato plants pretreated with azelaic acid

Gene name	Function	Accession number	Primer sequences	Amplification size (bp)	Reference
*SlNPR1*	Transcription factor	NM_001247629.2	F-GGGAAAGATAGCAGCACG	144	[52]
			R-TCCACACAAACACACACATC		
*SlERF2*	Transcription factor	NM_001347076.1	F-ACATTTGAATTTCCCGCACCG	135	Designed in this study
			R-TGAACGGCTTTTCTTCTCCGT		
*SlPR1*	Pathogenesis-related gene	NM_001247429.1	F-GGTAACTGGAGAGGACAACG	170	[58]
			R-GTCACATAAGCATAGCCTGG		
*SlPDF1.2*	Pathogenesis-related gene	NM_001247943	F-CTGGACCAATGAGAATTGTTG	112	[59]
			R-AATCCTTCGGTCCACATACC		
*SlActin*	Housekeeping gene	NM_001308447.1	F- AACAGACAGGACACTCGCACT	126	[60]
			R- TTAGCACCTTCCAGCAGATGT		

#### Statistical analysis

The experiments were conducted as a factorial experiment (2 × 2, pathogen inoculation and non-inoculation × treatment and non-treatment) for each time course. Additionally, two technical replicates were performed for biochemical and molecular tests. The least significant difference (LSD) test was performed at a 1% probability level (*P* < 0.01) for the analysis of mean comparison of growth characters. The comparison of biochemical assays was performed using the Scott & Knott test (*P* < 0.05). All statistical analyses were carried out using the InfoStat version. 2018 [36].

## Results

### Disease intensity analysis

**Fig. 1:**
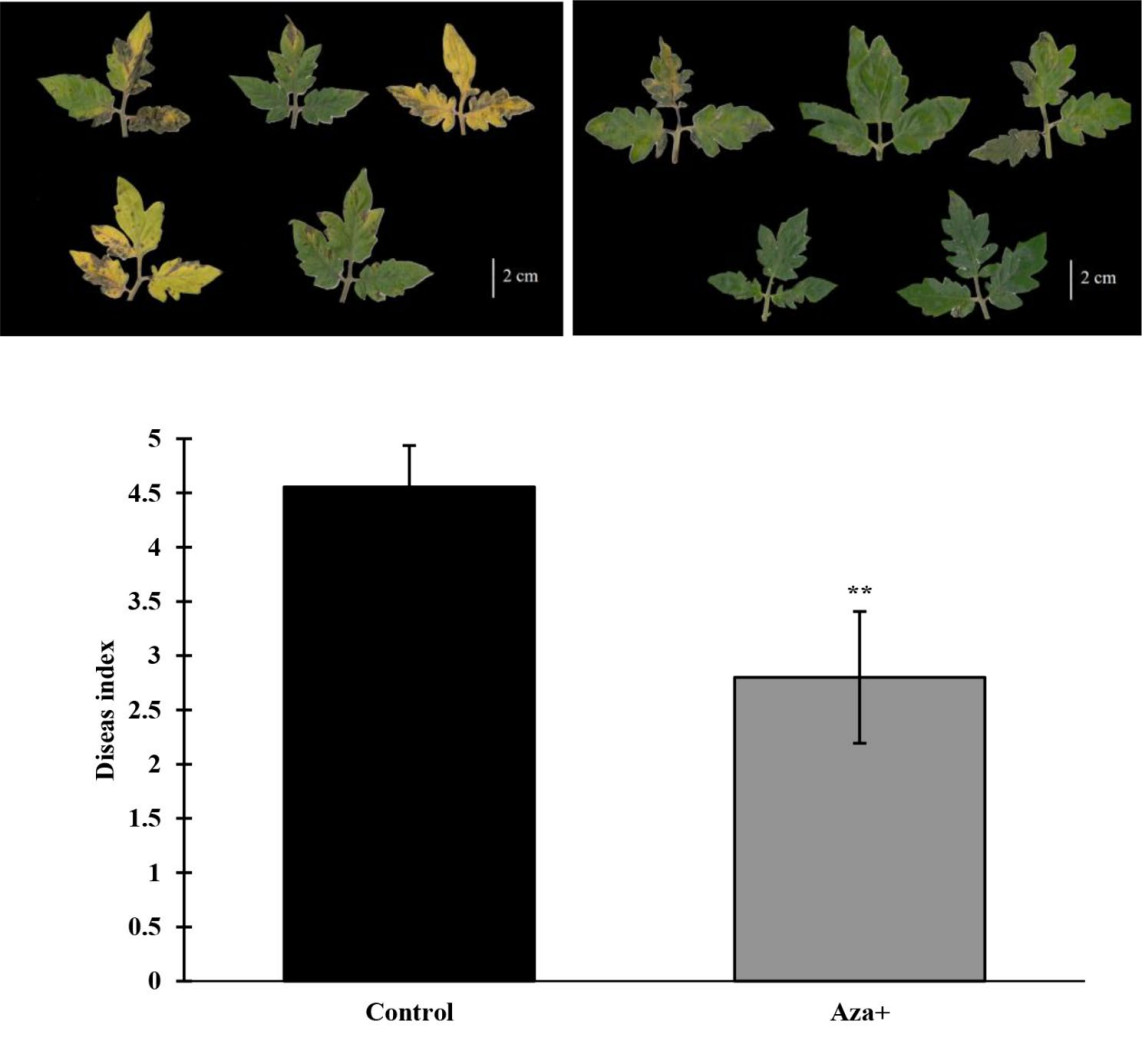
Disease index of azelaic acid treated (Aza+) and untreated (Control) tomato plants under *A. solani* stress in greenhouse conditions 21 days inoculation. At least 30 plants were investigated for each treatment. The significant level was measured based on the T-test *p* ≤ 0.01

Disease symptom analysis revealed that azelaic acid treatment led to a significant decrease in the early blight disease index (DI). DI was reduced by 37.7% in the Aza + treatment compared to the C + plants. The disease index in the C + treatment (untreated plants) was about 4.5, while it was 2.8 in the Aza + treatment (Fig. 1). This result indicates that the severity of early blight in the control plants (C+) was significantly higher than in the plants treated with azelaic acid (Aza+). In addition, the observations indicated that the disease spots were larger, and the damage caused by pathogen infection was more severe in the C + treatment than in the Aza + treatment. It can be concluded that the application of azelaic acid had a positive effect on the number of disease spots and the spread of disease on tomato leaves.

### Plant fresh and dry weights

**Fig. 2:**
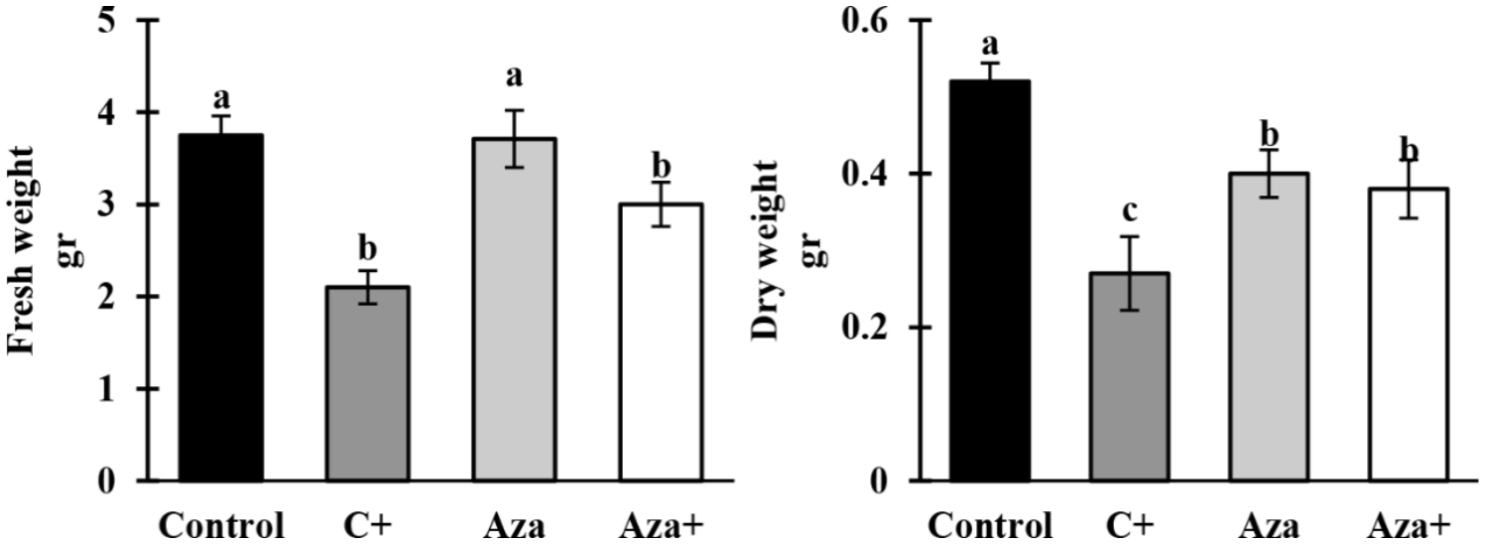
A comparison of fresh (FW) and dry (DW) weights of tomato plant shoot 21 days after inoculation with *A. solani* and pretreated with 1mM azelaic acid. Samples were allocated into four treatments as follows; Control (tomato seedlings received no treatment), C+ (tomato seedlings were inoculated with *A. solani*), Aza (tomato seedlings were treated with 1 mM azelaic acid), and Aza+ (tomato seedlings were treated with 1mM azelaic acid + inoculated with A. solani). Data show average values ± standard error (*n* = 6). The same letters are not significantly different according to the LSD test (*P* < 0.01)

Pathogen infection decreased fresh and dry weights of the C + plants by 44% and 48%, respectively, compared to control plants. Although, Aza application decreased plant dry weight in the absence of pathogen inoculation (Aza treatment), the dry weight of Aza-treated plants did not decrease significantly after pathogen inoculation (Aza+), despite a 23% decrease in their fresh weight. It was revealed that Aza treatment has mitigated the adverse effects of the disease on plant growth traits (Fig. 2).

#### Antioxidant enzyme activity

##### SOD enzyme activity

**Fig. 3:**
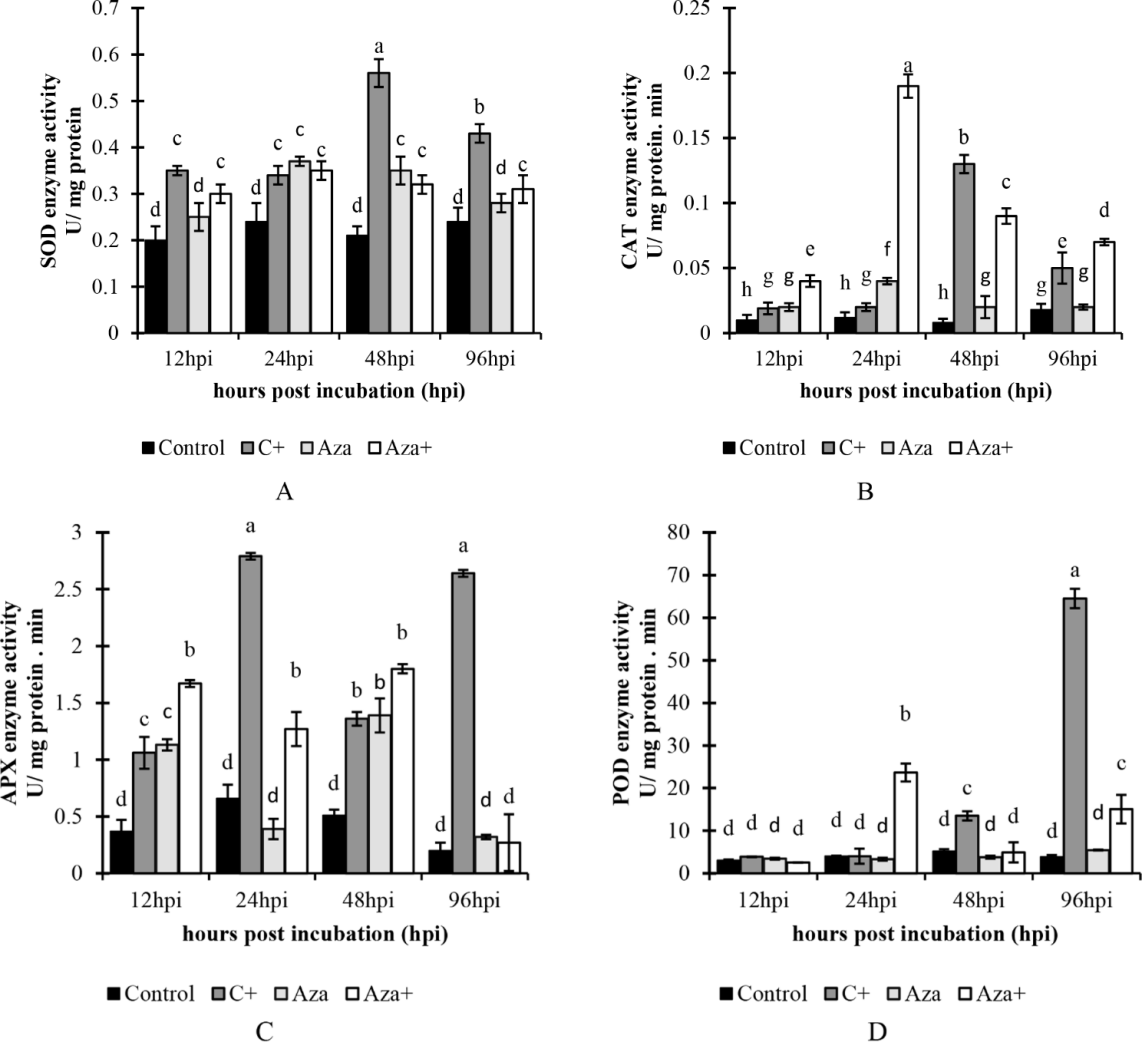
A comparison of (**A**) CAT, (**B**) SOD, (**C**) APX, and (**D**) POD enzyme activity over a 96-hour time course from 12 to 96 h after inoculation with *A. solani* and pretreated with 1mM azelaic acid. Samples were allocated into four treatments as follows; Control (tomato seedlings received no treatment), C+ (tomato seedlings were inoculated with *A. solani*), Aza (tomato seedlings were treated with 1 mM azelaic acid), and Aza+ (tomato seedlings were treated with 1mM azelaic acid + inoculated with *A. solani*). Data show average values ± standard error (*n* = 18). The same letters are not significantly different according to the Scott & Knott test (*P* < 0.05)

Superoxide dismutase (SOD) enzyme activity indicated that different treatments showed varying reactions to pathogen inoculation. Following pathogen inoculation in the C + treatment, the activity of the SOD enzyme gradually increased. It reached the maximum point (0.56 U/mg protein) at 48 hpi and then decreased at 96 hpi (0.43 U/mg protein), while its activity did not sharply increase in Aza and Aza + treatments. In the Aza treatment, enzyme activity was significantly increased 24- and 48 h post-inoculation, up to 0.35 U/mg protein. In the Aza + treatment, although SOD enzyme activity was significantly increased, the trend of enzyme activity remained constant during sampling times and was approximately 50% (0.37 U/mg protein) higher than the control at 24 hpi (Fig. 3A).

#### CAT enzyme activity

CAT enzyme activity assay revealed a significant difference among the treatments. At C+, CAT enzyme activity reached the highest level after 48 h post-inoculation (0.13 U/mg protein. min), while at Aza+, the maximum activity was observed at 24 hpi (0.19 U/mg protein. min). However, the highest CAT activity in Aza treatment was recorded at 24 hpi. It seems that the plants treated with azelaic acid (Aza and Aza+) exhibit a common trend, but the intensity of CAT enzymatic activity varies significantly (Fig. 3B).

##### APX enzyme activity

APX enzyme activity significantly increased in the C + treatment at all sampling times (12 hpi, 24 hpi, 48 hpi, and 96 hpi) compared to the control. The maximum level of APX enzyme activity was observed in the C + treatment at 24 hpi with 2.79 U/mg protein, which was 3.22 times higher than the control. In the Aza treatment, the enzyme activity significantly increased at 12 hpi and 48 hpi compared to the control. In the Aza + treatment, APX activity significantly increased at 12, 24, and 48 hpi compared to the control (Fig. 3C).

##### POD enzyme activity

Peroxidase enzyme activity assay results showed that, among all treatments, there was no significant difference before 12 hpi. However, the enzyme activity in the Aza + treatment was significantly increased at 24 hpi and 96 hpi, reaching 23.67 (490% higher than the control) and 15.06 U/mg protein.min respectively. Also, in the C + treatment, the enzyme activity was increased at 48 hpi and reached a maximum of 64.5 U/mg protein. min at 96 hpi. The treatment group showed a 16.88-fold increase compared to the control group at 96 hpi (Fig. 3D). The findings indicate that treatment with azelaic acid and pathogen infection enhanced the activity of the POD enzyme in tomato plants.

### Metabolite analysis

#### H_2_O_2_ content

**Fig. 4:**
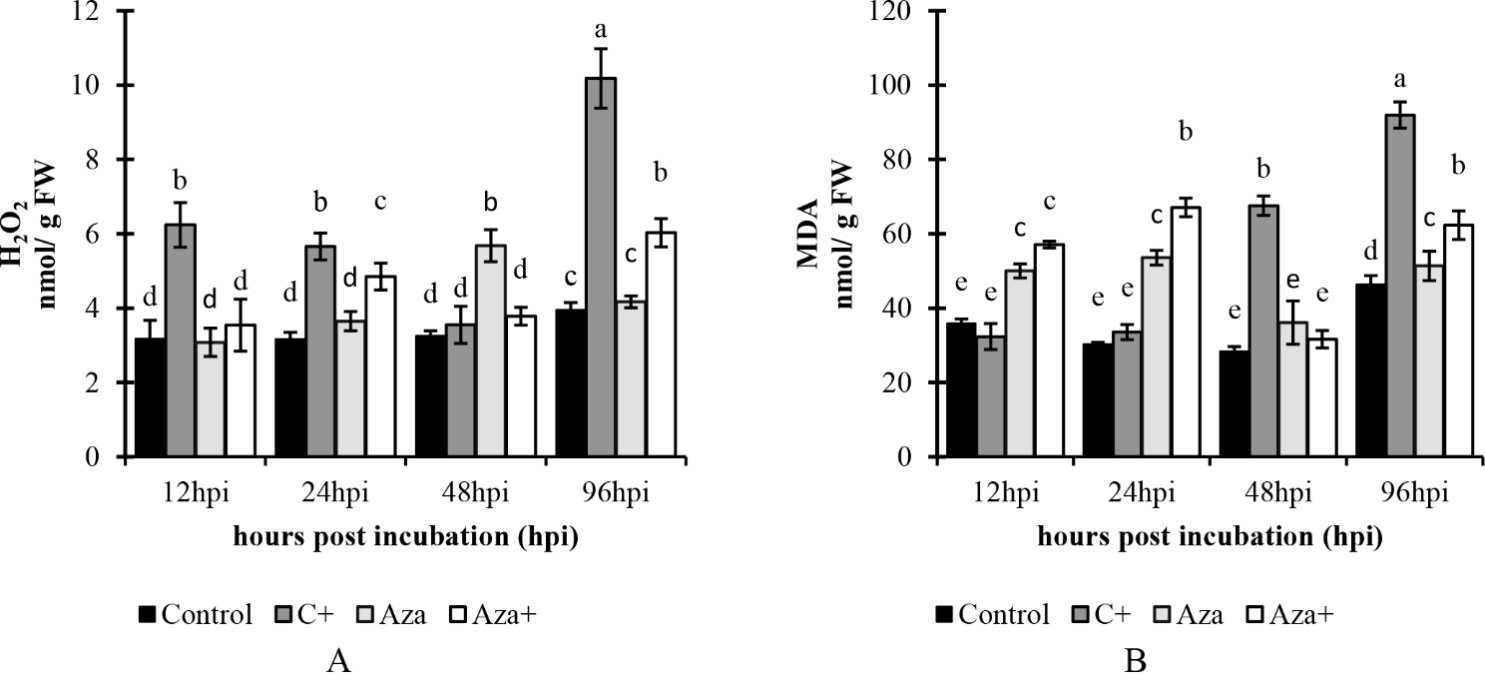
A comparison of (**A**) H_2_O_2_ and (**B**) MDA contents over a 96-hour time course from 12 to 96 h after inoculation with *A. solani* and pretreated with 1mM azelaic acid. Samples were allocated into four treatments as follows; Control (tomato seedlings received no treatment), C+ (tomato seedlings were inoculated with *A. solani*), Aza (tomato seedlings were treated with 1 mM azelaic acid), and Aza+ (tomato seedlings were treated with 1mM azelaic acid + inoculated with *A. solani*). Data show average values ± standard error (*n* = 18). The same letters are not significantly different according to the Scott & Knott test (*P* < 0.05)

Analysis of tomato leaves showed that H_2_O_2_ accumulation was significantly increased in the C + treatment at all-time courses except for 48 hpi. The highest level was observed at 96 hpi in the C + treatment, measuring 10.18 nmol/gFW (approximately 157.7% higher than the control plants). In the Aza treatment, there was a significant enhancement in H_2_O_2_ accumulation at 48 hpi, reaching 5.68 nmol/gFW (an increase of 74.76% compared to the control). In Aza + plants, the accumulation of H_2_O_2_ was significantly increased at 24 hpi and 96 hpi, reaching 4.85 nmol/gFW (an increase of 53.48% compared to the control) and 6.03 nmol/gFW (an increase of 52.65% compared to the control), respectively (Fig. 4A).

#### MDA content

MDA content was significantly increased in the C + at 48 hpi (67.57 nmol/gFW, 138.5% increase compared to the control group). At 96 hpi, it reached the maximum level of 91.97 nmol/gFW. In Aza treatment, significant accumulations of MDA were observed at 12 hpi, 24 hpi, and 96 hpi with values of 50.3, 53.57, and 51.37, respectively. Like the Aza treatment, the MDA accumulation level in the Aza + treatment was significantly altered at 12 hpi, 24 hpi, and 96 hpi, with values of 57.1, 67.1, and 62.33, respectively (Fig. 4B).

### Phytohormone analysis

#### Jasmonic acid

**Fig. 5:**
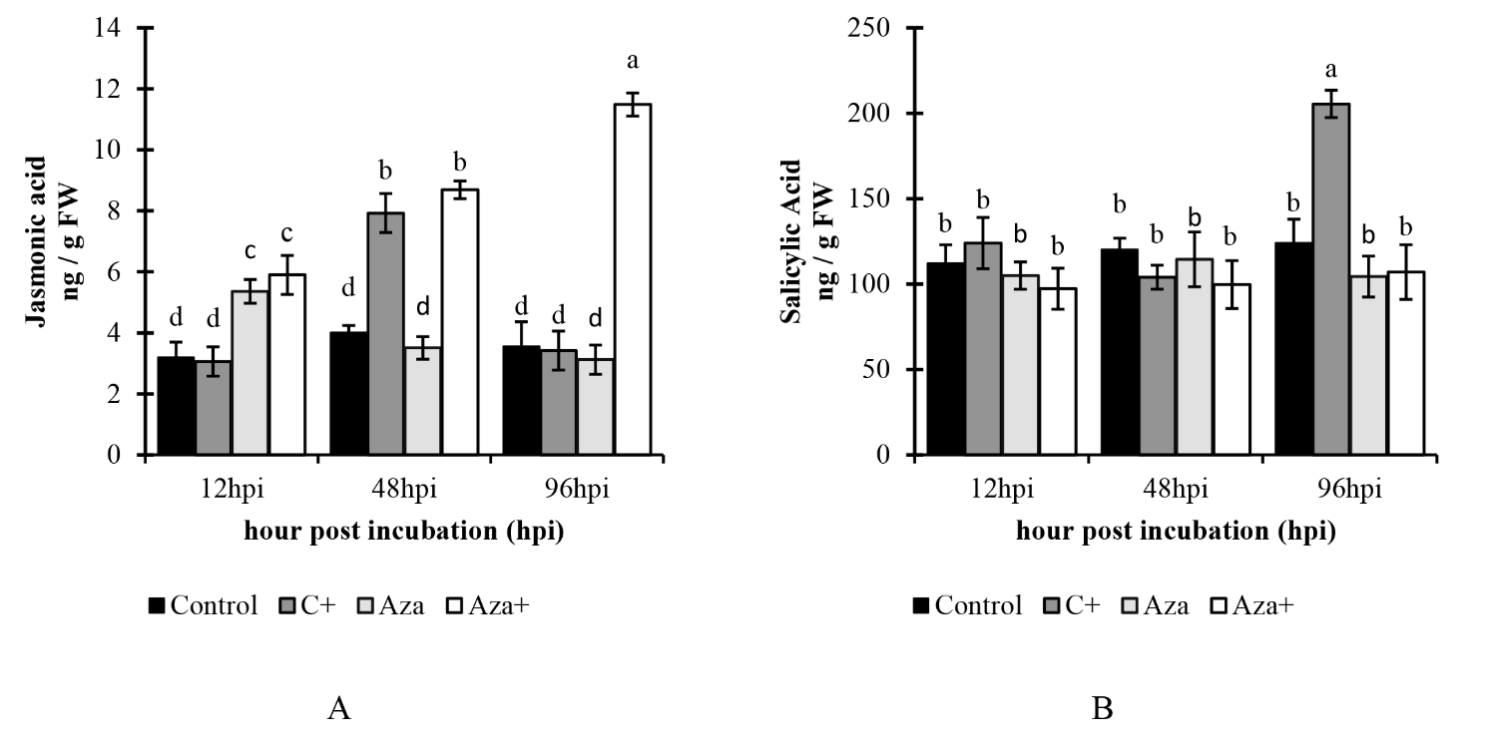
A comparison of (**A**) Jasmonic acid and (**B**) salicylic acid contents over a 96-hour time course from 12 to 96 h after inoculation with *A. solani* and pretreated with 1mM azelaic acid. Samples were allocated into four treatments as follows; Control (tomato seedlings received no treatment), C+ (tomato seedlings were inoculated with *A. solani*), Aza (tomato seedlings were treated with 1 mM azelaic acid), and Aza+ (tomato seedlings were treated with 1mM azelaic acid + inoculated with *A. solani*). Data show average values ± standard error (*n* = 18). The same letters are not significantly different according to the Scott & Knott test (*P* < 0.05)

Jasmonic acid analysis revealed that JA accumulation was increased in the C + treatment (around 7.92 ng/gFW) at 48 hpi. However, no significant difference was observed for the same treatment at 12 hpi and 96 hpi compared to the control. The level of jasmonic acid significantly increased by 67.7% (5.89 ng/gFW) in the Aza treatment compared to the control at 12 hpi. While the accumulation of JA in this treatment was not significantly different compared to the control at 48 hpi, and 96 hpi. In the Aza + treatment, JA accumulation increased steadily in all studied time courses. The highest level of JA in this treatment was recorded at 96 hpi for 11.48 ng/gFW (Fig. 5-A). Azelaic acid treatment appears to enhance the accumulation of jasmonic acid during the early stages following a pathogen attack.

#### Salicylic acid

Salicylic acid analysis showed that there was no significant difference among the treatments at 12 hpi and 48 hpi. However, the highest level of SA (205.47 ng/gFW) was observed in C + at 96 hpi. In contrast, the amount of this hormone did not show a significant change in Aza and Aza + treatments (Fig. 5-B).

### Gene expression analysis

#### SlNPR1

**Fig. 6:**
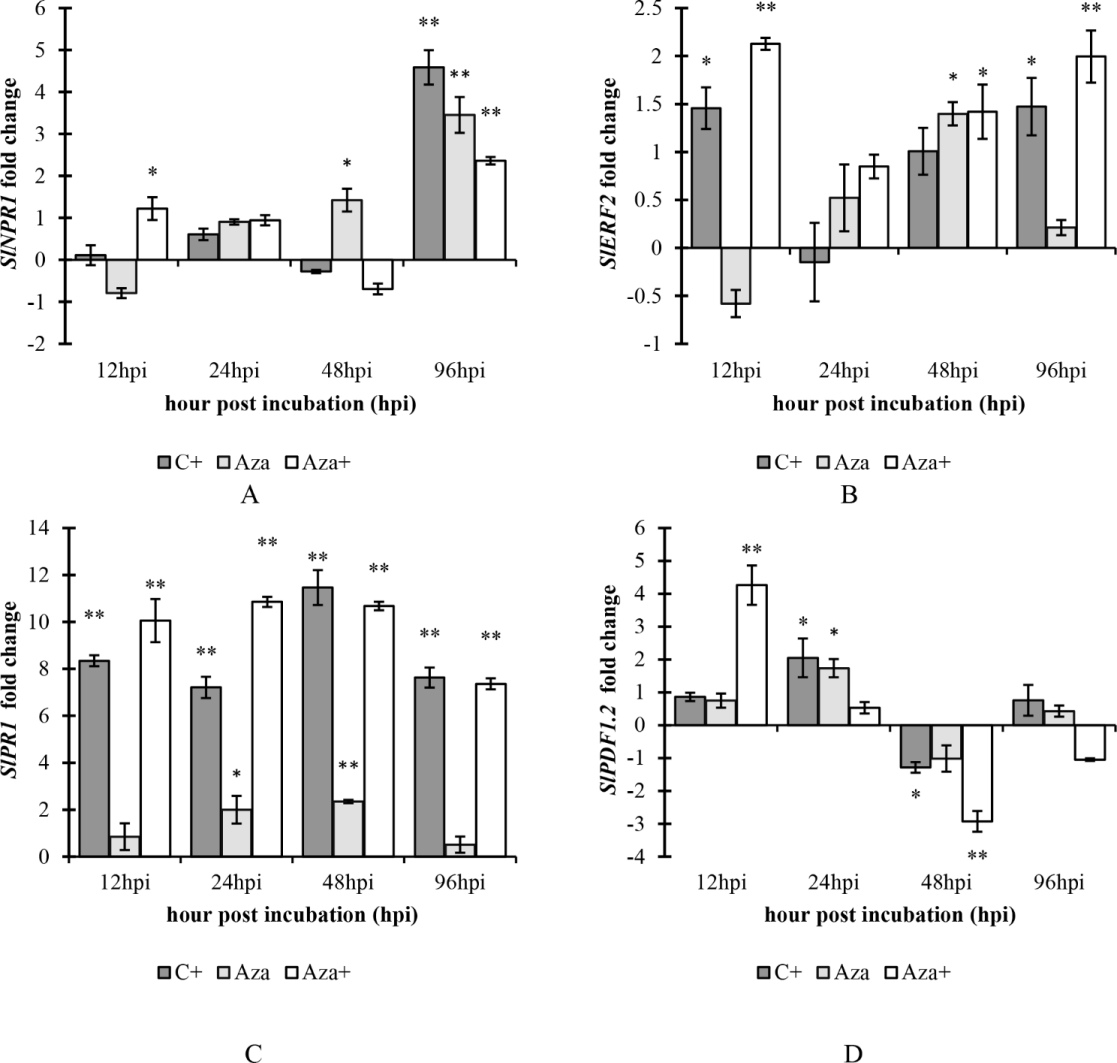
Relative gene expression profile of (**A**), *SlNPR1* (**B**), *SlERF2* (**C**), *SlPR1* and (**D**) *SlPDF1.2* gene over a 96-hour time course from 12 to 96 h after inoculation with *A. solani* and pretreated with 1mM azelaic acid. Samples were allocated into four treatments as follows; Control (tomato seedlings received no treatment), C+ (tomato seedlings were inoculated with *A. solani*), Aza (tomato seedlings were treated with 1 mM azelaic acid), and Aza+ (tomato seedlings were treated with 1mM azelaic acid + inoculated with *A. solani*). Data show average values ± standard error (*n* = 18). The same letters are not significantly different according to the T-test (* *P* < 0.05, ** *P* < 0.05)

The qRT-PCR assay revealed no significant transcript level in the C + treatment at all studied time courses, except at 96 hpi, where it increased by 4.58 times compared to the control. While in Aza treatment, the gene expression level significantly increased at 48 hpi and 96 hpi, with a 1.42-fold and 2.6-fold change, respectively. Based on the Aza + treatment, the expression of *SlNPR1* was up-regulated 1.22 and 2.36 times more than the control at 12 hpi and 96 hpi, respectively (Fig. 6A).

#### SlERF2

Gene expression analysis showed that the expression of *SIERF2* was different among the treatments (Fig. 5-A). C + treatment showed increased expression in the *SIERF2* transcript levels at 12hpi (1.45-fold change) and 96 hpi (1.47 times higher than the control). However, there was a significant increase (1.39 times higher than the control) in gene expression observed in the Aza treatment at 48 hpi. In the Aza + treatment, the expression of the *SlERF2* gene was up-regulated at 12 hpi, 48 hpi, and 96 hpi, showing 2.1, 1.4-, and 1.99-times higher expression compared to the control, respectively (Fig. 5-B).

### SlPR1

*SIPR1* transcript was induced in both the Aza + and C + treatments at all-time courses studied. The maximum up-regulation of gene expression was observed in the C + treatment at 48 hpi, which was 12 times higher than the control. However, the highest level of *SlPR1* transcript in the Aza + treatment was observed at 24 hpi, which was 10.85 times higher than the control. Related to the Aza treatment, a significant increase was observed at 24 hpi and 48 hpi, 1.9 and 2.3 times higher than the control, respectively (Fig. 6-C).

### SlPDF1.2

The expression of *SlPDF1.2* in C + treatment was significantly increased (approximately two times higher than the control) at 12 hpi, while its expression was decreased below the control plants (1.28 times lower than the control) at 24 hpi and was remained constant at 48 hpi. In the Aza treatment, the expression of *SlPDF1.2* was significantly altered only at 48 hpi, showing a 1.8-fold increase compared to the control. In the Aza + treatment, gene expression was up-regulated at 12 hpi and then down-regulated at 48 hpi. The levels of gene expression at 12 hpi and 48 hpi were 4.26-fold and − 3-fold higher than the control, respectively (Fig. 6-D).

## Discussion

In this study, we investigated the impact of exogenous azelaic acid on tomato plants infected with *A. solani* using various assays. Our results indicate that azelaic acid generally enhances defense reactions in *A. solani*-infected tomato plants, resulting in a significantly reduced disease index. Azelaic acid treatment induced distinct defense responses in tomato plants infected with *A. solani* compared to the control plants. While fungal infection was the main cause of changes in growth parameters, i.e., plant’s fresh and dry weights, azelaic acid treatment alleviated some of the negative effects caused by the pathogen infection. After being infected by pathogens, plants undergo significant physiological changes that ultimately lead to a decrease in biomass production and growth rate. There are several reports of a negative correlation between pathogen damage and the fresh and dry weights of plants [10].

The activity of ROS-scavenging enzymes in plant tissues generally changes during a pathogen attack [37]. The enhanced activity of antioxidant enzymes such as APX, CAT, and POD is a result of the accumulation of H_2_O_2_ in plant tissues. Rabiei et al. (2020) observed that the activity of antioxidant enzymes (APX and POD) and SOD gene expression in tomato plants increased after infection with *A. solani*. This increase was accompanied by an accumulation of H_2_O_2_. [16]. While it is believed that an increased antioxidant enzyme activity can improve a plant’s ability to defend against pathogens [38], it also increases in other stress conditions. In the current study, all studied antioxidant enzymes (SOD, APX, and POD), except for CAT, as well as H_2_O_2_ accumulation showed significantly higher activities in the C + plants compared to the Aza + plants. The significant difference in the activity of POD, APX, and SOD enzymes, as well as the accumulation of H_2_O_2_ in the C + treatment compared to Aza+, seems to have been resulted from more intense oxidative stress in C + treatment. Previous studies indicated that a balance of antioxidant enzymes such as SOD, APX, and POD is crucial for maintaining stable levels of H_2_O_2_ and O^− 2^, and for preventing damage caused by oxidative stress [16, 39].

Some catalase enzyme isoforms play important roles in plant resistance against necrotrophic pathogens by altering phytohormones balance. Zhang et al. (2021) illustrated that JA synthesis is induced by CAT2, which leads to increased resistance against necrotrophic *Botrytis cinerea*. In contrast, SA suppresses JA biosynthesis through inhibition of CAT2 activity [40]. In the present study, CAT enzyme activity was higher in the Aza + plants compared to C + plants.

Although cell death is one of the most efficient plant defense responses against pathogen infestation [41], this mechanism may also accelerate necrosis, which can make it easier for necrotrophic pathogens to infect plants. Studies have shown that after infiltration by necrotrophic pathogens, such as *A. solani*, the accumulation of hydrogen peroxide in host plant tissues increases, eventually leading to cell death [42, 43]. In this regard, Perchepied and et al. (2010) observed that some compounds such as oxalic acid, a key pathogenicity element, are elicitors of ROS production and programmed cell death in plants. They stated that plant infection by necrotrophic pathogens is facilitated by hypersensitive programmed cell death [44]. The accumulation of hydrogen peroxide in plant tissues may lead to cell death and several antioxidant enzymes have been evolved to prevent excessive accumulation of H_2_O_2_. Catalase, as one of the major antioxidant enzymes, converts H_2_O_2_ into H_2_O and O_2_ within the cell, thereby reducing the damage caused by H_2_O_2_ accumulation [45, 46]. The findings of the present study indicate that the accumulation of H_2_O_2_ increased over time in the C + treatment, while its content remained relatively low in Aza + treatment. Nafisa et al. (2020) reported that higher catalase activity is one of the most effective defense mechanisms in tomato plants against *A. solani*. In their study, 29 different tomato genotypes were evaluated and it was revealed that CAT enzyme activity was higher in the resistant cultivars compared to the susceptible ones [4].

MDA formation is a reliable indicator of peroxidation rate and can be used to predict the degradation ratio of cell membranes during exposure to ROS stress. It has been reported that pathogen infiltration and damage to plant tissues can lead to increased rates of lipid peroxidation [47]. In our study, we observed the highest MDA accumulation in the C + treatment after 96 h post-inoculation (hpi). This accumulation rate was approximately 32.2% higher than that of Aza + treatment at the same time.

In this study, the levels of MDA and DI were lower in the plants treated with azelaic acid compared to untreated plants infected with the pathogen. It seems that azelaic acid primarily reduced plant tissue damage via reduced H_2_O_2_ production upon pathogen attack. Similar results were reported by Noorbakhsh and Taheri (2016), who reported that nitric oxide (NO) application decreased the MDA content in tomato plants infected with *Rhizoctonia solani* mainly by reducing ROS levels [8].

It has been suggested that azelaic acid acts as a priming agent, enhancing the plant’s defense response upon pathogen attack [19]. In the current study, the transcript level of *SlNPR1* increased during the early stages of pathogen infection (12 hpi) in the Aza + treatment, while its expression was elevated 48 h after pathogen attack in the C + treatment. Several studies have indicated that *NPR1* regulates various defense mechanisms in plants [48–50]. While *NPR1* is recognized as a crucial element in regulating SA, Lai et al. (2018) demonstrated that *NPR1* expression depends on *NPR1*-inducing mechanisms that are independent of SA in response to stress. It was also revealed that these mechanisms are also utilized by a separate stress signaling pathway with distinct functions [50]. In the present study, although SA accumulation was not significantly changed in the Aza + treatment compared to the control, its content was increased in the C + treatment during the late stage of the pathogen attack (96 hpi). Generally, SA accumulation may lead to plant cell death as a defense mechanism during plant-pathogen interactions, which is particularly effective against biotrophic pathogens [51].

In contrast, Rahman et al. (2012) demonstrated that a necrotrophic pathogen, like *A. solani*, enhances the sensitivity of tomato plants by increasing the accumulation of SA and overexpression of *NPR1* gene [52]. The salicylic acid-related pathway serves as the primary defense mechanism against biotrophic pathogens. On the other hand, based on previous studies, azelaic acid is believed to trigger increased SA accumulation in plant tissues. The present study did not find a significant increase in SA accumulation with Aza + and Aza treatments. Jung et al. (2009) observed that azelaic acid treatment increased SA accumulation in in distant leaves of Arabidopsis plants upon inoculation with *P. syringae* pv. *maculicola strain Pma*DG3. Their findings revealed that exogenous application of azelaic acid per se could not enhance SA accumulation level [19]. It could support our finding in which SA did not significantly change upon Aza treatment. However, Yu et al. (2013) did not observe the priming impact of azelaic acid on SA accumulation in *Arabidopsis* plants. They reported that a higher accumulation of SA after Aza application requires increased levels of G3P (a phosphorylated sugar derivative of glycerol-3-phosphate) and high concentrations of reactive oxygen species (ROS) [18]. As previously mentioned in our study, compared to C + treatment, Aza + treatment exhibited a reduction of 32.2% in the rate of ROS-derived damage, as determined by the MDA index. It seems that this could be the reason for the lack of a significant increase in SA in Aza + treatment.

The impact of inducers on plant defense system can affected by several important factors such as plant species, type of inducer, and pathogen. For example, the effect of azelaic acid on on the defense responses of *Arabidopsis* plants was reported by Jung et al. (2009) and Yu et al. (2013), while an study conducted by Nagy et al. (2017) showed that treatment with azelaic acid did not induce tobacco plants defense system against viral and bacterial pathogens. Their study indicated that, although previous studies suggested azelaic acid as a signal transduction element for SAR in *Arabidopsis*, its role cannot be confirmed in tobacco plants [53]. Therefore, the exact effect of a substance such as azelaic acid would be prone to alterations in different experiments involving different plants and pathogens.

During pathogen stress, the crosstalk between salicylic acid (SA) and jasmonic acid (JA) is crucial in plant defense responses. It is generally accepted that SA-mediated defense response plays a central role in local and systemic-acquired resistance (SAR) against biotrophic pathogens. In contrast, the ET/JA-mediated response contributes to defense against necrotrophic pathogens. These two pathways are mutually antagonistic, meaning they have opposing effects on plant defense [54]. In the present study, the comparison of hormonal changes in C + treatment showed that at 48 hpi, JA concentration was increased significantly while SA content did not change significantly compared to the control. Meanwhile, as SA increases at 96 hpi, JA levels decrease. On the other hand, in the Aza + treatment, no significant difference was observed in SA accumulation, while, JA showed a constant increase in all the studied times.

Classically, the jasmonic acid-related defense system is the primary resistance pathway associated with necrotrophic pathogens [55]. However, some studies have suggested that resistance against necrotrophic pathogens, such as *A. solani*, also requires activation of SA signaling pathway [52, 56]. Liu et al. (2016) demonstrated that SA and JA hormones play important roles in plant responses to necrotrophic pathogens. They mentioned that as plants are often invaded by both necrotrophic and biotrophic pathogens, it is crucial to elevate the levels of both SA and JA [57]. In the present study, the expression of the *PDF1.2* gene, a marker for JA signaling, and the accumulation of JA in tissues were increased during the early stages of pathogen attack (12 hpi) in Aza + treatment. However, in C + treatment, the expression of *PDF1.2* was up-regulated at 24 hpi, resulting in increased JA accumulation at 48 hpi. Djami-Tchatchou et al. (2017) demonstrated that treatment with azelaic acid induced the expression of the *PDF1.2* gene in tobacco plants [20]. Our findings suggest that azelaic acid effects in mitigating *A. solani* damage may act by modulating both the SA and JA defense-related pathways.

## Conclusion

**Fig. 7:**
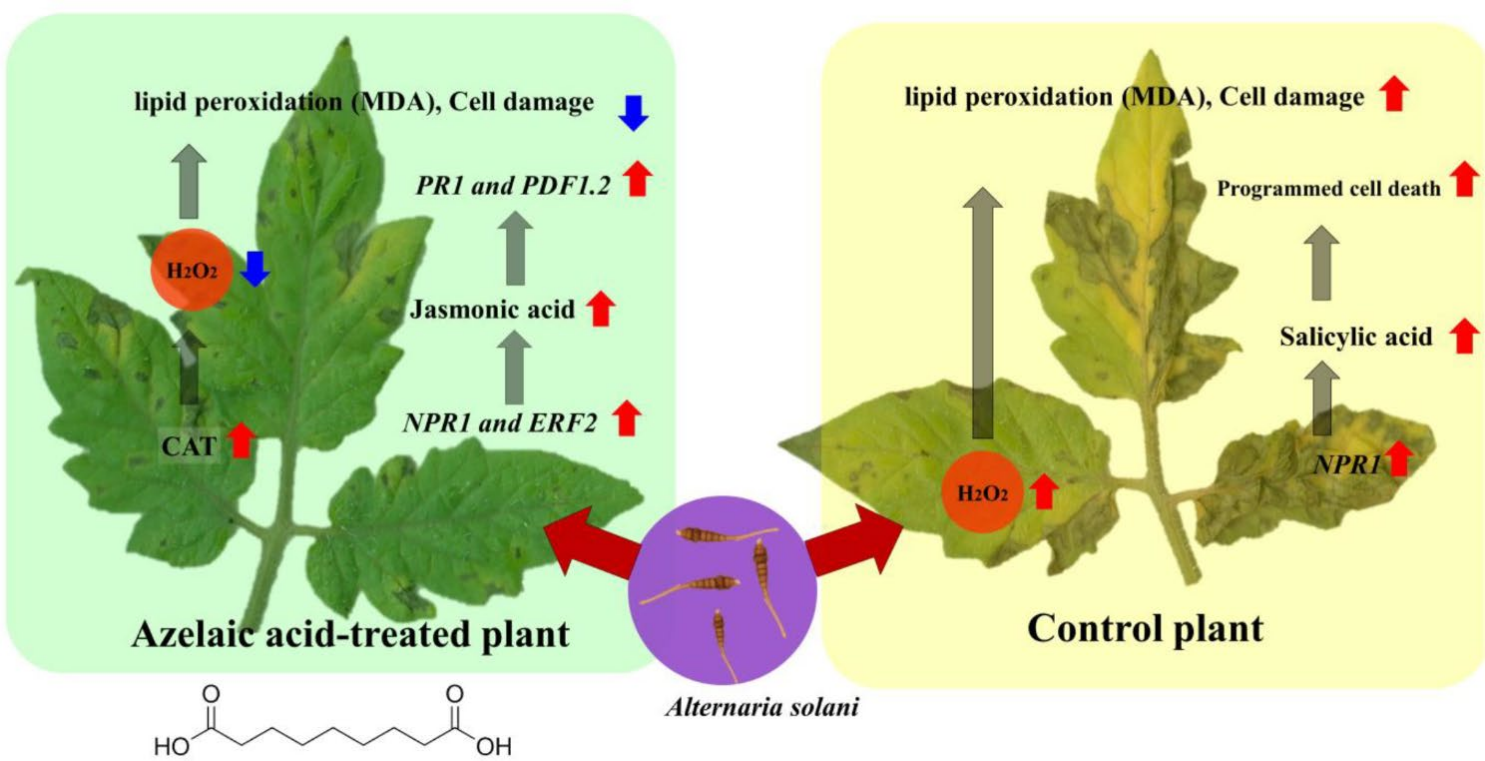
Schematic representation of tomato response to *Alternaria solani* infection as influenced by azelaic acid treatment. In control plant, ROS production and H_2_O_2_ accumulation were increased after pathogen attack resulting in increased cell wall lipid peroxidation. Enhanced *NPR1* gene expression increased Salicylic acid content increasing programmed cell death (MDA) leading to increased disease symptoms. Azelaic acid application reduced ROS content and decreased cell wall lipid peroxidation rate (MDA) by advanced catalase enzyme activity following pathogen infection. Azelaic acid treatment also increased expression of *NPR1* and *ERF2* genes, which in turn increased the accumulation of Jasmonic acid and induced *PR1* and *PDF1.2* expression after *A. solani attack*

The results suggest that azelaic acid application can mitigate the damage caused by *A. solani* as a necrotrophic pathogen in tomato plants. Surprisingly, SA accumulation was not significantly changed upon azelaic acid treatment in pathogen-infected plants. Furthermore, biochemical and molecular analyses imply that azelaic acid exerts its effects by modulating the ROS pathway, primarily by enhancing catalase enzyme activity leading to a reduced pathogen infection rate. Interestingly, azelaic acid not only increased the expression of the *NPR1* gene, which serves as a marker for SA signaling pathway, but also caused a significant increase in *PDF1.2* gene expression, a marker for the jasmonic acid (JA)/ethylene (ET) pathway, accompanied by higher accumulation of JA in plant tissues. It seems that azelaic acid-induced resistance may be a result of a complex interplay of antioxidant, phytohormonal, and molecular mechanisms in tomato plants during *A. solani* infection (Fig. 7).

## Data Availability

The datasets generated during and/or analyzed during the current study are available from the corresponding author upon reasonable request.

## Abbreviations

Aza: Azelaic acid
POD: Peroxidase
CAT: Catalase
SOD: Superoxide dismutase
APX: Ascorbate peroxidase
JA/ET: Jasmonic acid/ethylene
SA: Salicylic acid
SAR: Systemic acquired resistance
ISR: Induced systemic resistance
ROS: Reactive oxygen species

## References

[1] Li R, Sheng J, Shen L. Nitric oxide plays an important role in β-aminobutyric acid-induced resistance to *Botrytis Cinerea* in Tomato plants. Plant Pathol J. 2020;36:121–32. 10.5423/PPJ.OA.11.2019.0274. 32296292 10.5423/PPJ.OA.11.2019.0274 PMC7143515

[2] Gong C, Liu Y, Liu S, Cheng M, Zhang Y, Wang R, et al. Analysis of *Clonostachys Rosea*-induced resistance to grey mould disease and identification of the key proteins induced in tomato fruit. Postharvest Biol Technol. 2017;123:83–93. 10.1016/j.postharvbio.2016.08.004. 10.1016/j.postharvbio.2016.08.004

[3] Panthee D, Chen F. Genomics of fungal disease resistance in tomato. Curr Genomics. 2010;11:30–9. 10.2174/138920210790217927. 20808521 10.2174/138920210790217927 PMC2851114

[4] Nafisa SA, Iqbal J, Khan KA. Evaluation of phenotypic, physiological and biochemical attributes connected with resistance in tomato against *Alternaria solani*. Acta Physiol Plant. 2020;42:88. 10.1007/s11738-020-03076-2. 10.1007/s11738-020-03076-2

[5] Adhikari P, Oh Y, Panthee DR. Current status of early blight resistance in tomato: an update. Int J Mol Sci. 2017;18:2019. 10.3390/ijms18102019. 28934121 10.3390/ijms18102019 PMC5666701

[6] Machinandiarena MF, Lobato MC, Feldman ML, Daleo GR, Andreu AB. Potassium phosphite primes defense responses in potato against *Phytophthora infestans*. J Plant Physiol. 2012;169:1417–24. 10.1016/j.jplph.2012.05.005. 22727804 10.1016/j.jplph.2012.05.005

[7] Aranega-Bou P, de la Leyva O, Finiti M, Garcfa-Agustfn I, Gonzalez-Bosch P. Priming of plant resistance by natural compounds. Hexanoic acid as a model. Front Plant Sci. 2014;5 OCT:1–12. 10.3389/fpls.2014.00488. 10.3389/fpls.2014.00488 PMC4181288 25324848

[8] Noorbakhsh Z, Taheri P. Nitric oxide: a signaling molecule which activates cell wall-associated defense of tomato against *Rhizoctonia solani*. Eur J Plant Pathol. 2016;144:551–68. 10.1007/s10658-015-0794-5. 10.1007/s10658-015-0794-5

[9] Percival GC, Banks JM. Evaluation of plant defence activators for the potential control of *Pseudomonas syringae* Pv. *Aesculi*. Arboric J. 2014;36:76–88. 10.1080/03071375.2014.921396. 10.1080/03071375.2014.921396

[10] Mofidnakhaei M, Abdossi V, Dehestani A, Pirdashti H, Babaeizad V. Potassium phosphite affects growth, antioxidant enzymes activity and alleviates disease damage in cucumber plants inoculated with *Pythium ultimum*. Arch Phytopathol Plant Prot. 2016;49:207–21. 10.1080/03235408.2016.1180924. 10.1080/03235408.2016.1180924

[11] Ramezani M, Rahmani F, Dehestani A. Study of physio-biochemical responses elicited by potassium phosphite in downy mildew-infected cucumber plants. Arch Phytopathol Plant Prot. 2017;50:540–54. 10.1080/03235408.2017.1341140. 10.1080/03235408.2017.1341140

[12] Ramezani M, Ramezani F, Rahmani F, Dehestani A. Exogenous potassium phosphite application improved PR-protein expression and associated physio-biochemical events in cucumber challenged by *Pseudoperonospora Cubensis*. Sci Hortic. 2018;234:335–43. 10.1016/j.scienta.2018.02.042. 10.1016/j.scienta.2018.02.042

[13] Zhou C, Zhu J, Qian N, Guo J, Yan C. Bacillus subtilis SL18r induces tomato resistance against Botrytis Cinerea, involving activation of long non-coding RNA, MSTRG18363, to decoy miR1918. Front Plant Sci. 2021;11:634819. 10.3389/fpls.2020.634819. 33613592 10.3389/fpls.2020.634819 PMC7887324

[14] Junglee S, Urban L, Sallanon H, Lopez-Lauri F. Optimized assay for hydrogen peroxide determination in plant tissue using potassium iodide. Am J Anal Chem. 2014;05:730–6. 10.4236/ajac.2014.511081. 10.4236/ajac.2014.511081

[15] Chen Y, Bendix C, Lewis JD. Comparative genomics screen identifies microbe-associated molecular patterns from ‘ *Candidatus* Liberibacter’ spp. that elicit immune responses in plants. mol plant-microbe Interact. 2020;33:539–52. 10.1094/MPMI-11-19-0309-R. 31790346 10.1094/MPMI-11-19-0309-R

[16] Rabiei Z, Hosseini S, Dehestani A, Pirdashti H, Beiki F. Exogenous hexanoic acid induced primary defense responses in tomato (*Solanum lycopersicum* L.) plants infected with *Alternaria solani*. Sci Hortic. 2022;295:110841. 10.1016/j.scienta.2021.110841. 10.1016/j.scienta.2021.110841

[17] Shah J, Zeier J. Long-distance communication and signal amplification in systemic acquired resistance. Front Plant Sci. 2013;4:30. 10.3389/fpls.2013.00030. 23440336 10.3389/fpls.2013.00030 PMC3579191

[18] Yu K, Soares JM, Mandal MK, Wang C, Chanda B, Gifford AN, et al. A feedback regulatory loop between G3P and lipid transfer proteins DIR1 and AZI1. Cell Rep. 2013;3:1266–78. 10.1016/j.celrep.2013.03.030. 23602565 10.1016/j.celrep.2013.03.030

[19] Jung W, Tschaplinski TJ, Wang L, Glazebrook J, Greenberg JT. Priming in systemic plant immunity. Sci (80). 2009;324:89–91. 10.1126/science.1170025. 10.1126/science.1170025 19342588

[20] Djami-Tchatchou AT, Ncube EN, Steenkamp PA, Dubery IA. Similar, but different: structurally related azelaic acid and hexanoic acid trigger differential metabolomic and transcriptomic responses in tobacco cells. BMC Plant Biol. 2017;17:227. 10.1186/s12870-017-1157-5. 29187153 10.1186/s12870-017-1157-5 PMC5706331

[21] Hoagland DR, Arnon DI. The water-culture method for growing plants without soil. Circ Calif Agric Exp Stn. 1950;347 2nd edit.

[22] Ramezani Y, Taheri P, Mamarabadi M. Identification of *Alternaria* spp. associated with tomato early blight in Iran and investigating some of their virulence factors. J Plant Pathol. 2019;101:647–59. 10.1007/s42161-019-00259-w. 10.1007/s42161-019-00259-w

[23] Laflamme B, Middleton M, Lo T, Desveaux D, Guttman DS. Image-based quantification of plant immunity and disease. Mol Plant-Microbe Interact. 2016;29:919–24. 10.1094/MPMI-07-16-0129-TA. 27996374 10.1094/MPMI-07-16-0129-TA

[24] Pandey KK, Pandey PK, Kalloo G, Banerjee MK. Resistance to early blight of tomato with respect to various parameters of disease epidemics. J Gen Plant Pathol. 2003;69:364–71. 10.1007/s10327-003-0074-7. 10.1007/s10327-003-0074-7

[25] Rodríguez-Rosales MP, Kerkeb L, Bueno P, Donaire JP. Changes induced by NaCl in lipid content and composition, lipoxygenase, plasma membrane H+-ATPase and antioxidant enzyme activities of tomato (*Lycopersicon esculentum*. Mill) calli. Plant Sci. 1999;143:143–50. 10.1016/S0168-9452(99)00046-1. 10.1016/S0168-9452(99)00046-1

[26] Esfahani L, Babaeizad VHR, Dehestani A. Alterations in antioxidant enzyme activities in rice plants treated with various abiotic inducers against the bacterial blight agent *Xanthomonas oryzae* Pv. Oryzae. J Plant Mol Breed. 2023;11:41–53. 10.22058/JPMB.2024.2010427.1284. 10.22058/JPMB.2024.2010427.1284

[27] Bradford MM. A rapid and sensitive method for the quantitation of microgram quantities of protein utilizing the principle of protein-dye binding. Anal Biochem. 1976;72:248–54. 10.1016/0003-2697(76)90527-3. 942051 10.1016/0003-2697(76)90527-3

[28] Beauchamp C, Fridovich I. Superoxide dismutase: improved assays and an assay applicable to acrylamide gels. Anal Biochem. 1971;44:276–87. 10.1016/0003-2697(71)90370-8. 4943714 10.1016/0003-2697(71)90370-8

[29] Aebi H. Catalase. In: Methods of Enzymatic Analysis. 1974. pp. 673–84.

[30] Nakano Y, Asada K. Purification of ascorbate peroxidase in spinach chloroplasts; its inactivation in ascorbate-depleted medium and reactivation by monodehydroascorbate radical. Plant Cell Physiol. 1987;28:131–40. 10.1093/oxfordjournals.pcp.a077268. 10.1093/oxfordjournals.pcp.a077268

[31] Putter J. Peroxidases. Methods of enzymatic analysis. Elsevier; 1974. pp. 685–90. 10.1016/b978-0-12-091302-2.50033-5.

[32] Ghorbani A, Razavi SM, Omran VOG, Pirdashti H. *Piriformospora indica* alleviates salinity by boosting redox poise and antioxidative potential of tomato. Russ J Plant Physiol. 2018;65:898–907. 10.1134/S1021443718060079

[33] Ohkawa H, Ohishi N, Yagi K. Assay for lipid peroxides in animal tissues by thiobarbituric acid reaction. Anal Biochem. 1979;95:351–8. 10.1016/0003-2697(79)90738-3. 36810 10.1016/0003-2697(79)90738-3

[34] Alexieva V, Sergiev I, Mapelli S, Karanov E. The effect of drought and ultraviolet radiation on growth and stress markers in pea and wheat. Plant Cell Environ. 2001. 10.1046/j.1365-3040.2001.00778.x. 10.1046/j.1365-3040.2001.00778.x

[35] Livak KJ, Schmittgen TD. Analysis of relative gene expression data using real-time quantitative PCR and the 2^–∆∆CT^ method. Methods. 2001;25:402–8. 10.1006/meth.2001.1262. 11846609 10.1006/meth.2001.1262

[36] Di Rienzo JA, Casanoves F, Balzarini MG, Gonzalez L, Tablada M, Robledo CW. InfoStat version 2018 [Computer software]. Faculty of Agricultural Sciences, National University of Córdoba; 2018.

[37] Dumanovic J, Nepovimova E, Natic M, Kuca K, Jacevic V. The significance of reactive oxygen species and antioxidant defense system in plants: a concise overview. Front Plant Sci. 2021;11:552969. 10.3389/fpls.2020.552969. 33488637 10.3389/fpls.2020.552969 PMC7815643

[38] Feng L, Sun J, Jiang Y, Duan X. Role of reactive oxygen species against pathogens in relation to postharvest disease of papaya fruit. Horticulturae. 2022;8:205. 10.3390/horticulturae8030205. 10.3390/horticulturae8030205

[39] Li Y, Gu Y, Li J, Xu M, Wei Q, Wang Y. Biocontrol agent Bacillus amyloliquefaciens LJ02 induces systemic resistance against cucurbits powdery mildew. Front Microbiol. 2015;6:883. 10.3389/fmicb.2015.00883. 26379654 10.3389/fmicb.2015.00883 PMC4551870

[40] Zhang Y, Song R, Yuan H, Li T, Wang L, Lu K, et al. Overexpressing the N-terminus of CATALASE2 enhances plant jasmonic acid biosynthesis and resistance to necrotrophic pathogen *Botrytis Cinerea* B05.10. Mol Plant Pathol. 2021;22:1226–38. 10.1111/mpp.13106. 34247446 10.1111/mpp.13106 PMC8435237

[41] Pietrowska E, Różalska S, Kaźmierczak A, Nawrocka J, Małolepsza U. Reactive oxygen and nitrogen (ROS and RNS) species generation and cell death in tomato suspension cultures—*Botrytis Cinerea* interaction. Protoplasma. 2015;252:307–19. 10.1007/s00709-014-0680-6. 25064634 10.1007/s00709-014-0680-6 PMC4287684

[42] De Jong A, Yakimova E, Kapchina V, Woltering E. A critical role for ethylene in hydrogen peroxide release during programmed cell death in tomato suspension cells. Planta. 2002;214:537–45. 10.1007/s004250100654. 11925037 10.1007/s004250100654

[43] Wang Y, Lin A, Loake GJ, Chu C. H_2_O_2_-induced leaf cell death and the crosstalk of reactive Nitric/Oxygen species. J Integr Plant Biol. 2013;55:202–8. 10.1111/jipb.12032. 23331502 10.1111/jipb.12032

[44] Perchepied L, Balagué C, Riou C, Claudel-Renard C, Rivière N, Grezes-Besset B, et al. Nitric oxide participates in the complex interplay of defense-related signaling pathways controlling disease resistance to sclerotinia sclerotiorum in Arabidopsis thaliana. Mol Plant-Microbe Interact. 2010;23:846–60. 10.1094/MPMI-23-7-0846. 20521948 10.1094/MPMI-23-7-0846

[45] Moghaieb REA, Ahmed DS, Gaber A, Abdelhadi AA. Overexpression of bacterial *katE* gene improves the resistance of modified tomato plant against *Fusarium oxysporum* f. sp. *lycopersici*. GM Crops Food. 2021;12:315–27. 10.1080/21645698.2021.1903374. 10.1080/21645698.2021.1903374 PMC8018384 33783318

[46] Reitz NF, Mitcham EJ. Lignification of tomato (*Solanum lycopersicum*) pericarp tissue during blossom-end rot development. Sci Hortic. 2021;276:109759. 10.1016/j.scienta.2020.109759. 10.1016/j.scienta.2020.109759

[47] Mishra S, Jha AB, Dubey RS. Arsenite treatment induces oxidative stress, upregulates antioxidant system, and causes phytochelatin synthesis in rice seedlings. Protoplasma. 2011;248:565–77. 10.1007/s00709-010-0210-0. 20857150 10.1007/s00709-010-0210-0

[48] Innes R. The positives and negatives of NPR: a unifying model for salicylic acid signaling in plants. Cell. 2018;173:1314–5. 10.1016/j.cell.2018.05.034. 29856948 10.1016/j.cell.2018.05.034

[49] Ji C, Yang W, Wang Y, Su C, Li X, Liu P, et al. Key residues for maintaining architecture, assembly of plant hormone SA receptor NPR1. Biochem Biophys Res Commun. 2022;613:94–9. 10.1016/j.bbrc.2022.04.119. 35550200 10.1016/j.bbrc.2022.04.119

[50] Lai Y-S, Renna L, Yarema J, Ruberti C, He SY, Brandizzi F. Salicylic acid-independent role of NPR1 is required for protection from proteotoxic stress in the plant endoplasmic reticulum. Proc Natl Acad Sci. 2018;115:5203–12. 10.1073/pnas.1802254115. 10.1073/pnas.1802254115 PMC5984531 29760094

[51] Brodersen P, Malinovsky FG, Heematy K, Newman M-A, Mundy J. The role of salicylic acid in the induction of cell death in Arabidopsis acd11. Plant Physiol. 2005;138:1037–45. 10.1104/pp.105.059303. 15923330 10.1104/pp.105.059303 PMC1150418

[52] Rahman TA, El OM, El, Gonzalez-Lamothe R, Bouarab K. Necrotrophic pathogens use the salicylic acid signaling pathway to promote disease development in tomato. Mol Plant-Microbe Interact. 2012;25:1584–93. 10.1094/MPMI-07-12-0187-R. 22950753 10.1094/MPMI-07-12-0187-R

[53] Nagy Z, Kátay G, Gullner G, Király L, Ádám AL. Azelaic acid accumulates in phloem exudates of TMV-infected tobacco leaves, but its application does not induce local or systemic resistance against selected viral and bacterial pathogens. Acta Physiol Plant. 2017. 10.1007/s11738-016-2303-7. 10.1007/s11738-016-2303-7

[54] Li N, Han X, Feng D, Yuan D, Huang L-J. Signaling crosstalk between salicylic acid and ethylene/jasmonate in plant defense: do we understand what they are whispering? Int J Mol Sci. 2019;20:671. 10.3390/ijms20030671. 30720746 10.3390/ijms20030671 PMC6387439

[55] Koley P, Brahmachari S, Saha A, Deb C, Mondal M, Das N, et al. Phytohormone priming of tomato plants evoke differential behavior in *Rhizoctonia solani* during infection, with salicylate priming imparting greater tolerance than jasmonate. Front Plant Sci. 2022;12:766095. 10.3389/fpls.2021.766095. 35082805 10.3389/fpls.2021.766095 PMC8784698

[56] El Oirdi M, El Rahman TA, Rigano L, El Hadrami A, Rodriguez MC, Daayf F, et al. *Botrytis Cinerea* manipulates the antagonistic effects between immune pathways to promote disease development in tomato. Plant Cell. 2011;23:2405–21. 10.1105/tpc.111.083394. 21665999 10.1105/tpc.111.083394 PMC3160041

[57] Liu L, Sonbol F-M, Huot B, Gu Y, Withers J, Mwimba M, et al. Salicylic acid receptors activate jasmonic acid signalling through a non-canonical pathway to promote effector-triggered immunity. Nat Commun. 2016;7:13099. 10.1038/ncomms13099. 27725643 10.1038/ncomms13099 PMC5062614

[58] Moghaddam GA, Rezayatmand Z, Nasr Esfahani M, Khozaei M. Genetic defense analysis of tomatoes in response to early blight disease, *Alternaria alternata*. Plant Physiol Biochem. 2019;142:500–9. 10.1016/j.plaphy.2019.08.011. 31445475 10.1016/j.plaphy.2019.08.011

[59] Satkova P, Stary T, Pleskova V, Zapletalova M, Kasparovsky T, Cincalova-Kubienova L, et al. Diverse responses of wild and cultivated tomato to BABA, oligandrin and *oidium neolycopersici* infection. Ann Bot. 2016;119:829–40. 10.1093/aob/mcw188. 10.1093/aob/mcw188 PMC5378190 27660055

[60] VanDoorn A, De Vries M, Kant MR, Schuurink RC. Whiteflies glycosylate salicylic acid and secrete the conjugate via their honeydew. J Chem Ecol. 2015;41:52–8. 10.1007/s10886-014-0543-9. 25563984 10.1007/s10886-014-0543-9 PMC4303718

